# Roots and Leaf Extracts of *Dipsacus fullonum* L. and Their Biological Activities

**DOI:** 10.3390/plants9010078

**Published:** 2020-01-08

**Authors:** Jan Oszmiański, Aneta Wojdyło, Piotr Juszczyk, Paulina Nowicka

**Affiliations:** 1 Department of Fruit, Vegetable and Plant Nutraceuticals Technology, Wrocław University of Environmental and Life Sciences, 37 Chełmońskiego Street, 51-630 Wrocław, Poland; jan.oszmianski@upwr.edu.pl (J.O.); paulina.nowicka@upwr.edu.pl (P.N.); 2 Department of Biotechnology and Food Microbiology, Wrocław University of Environmental and Life Sciences, 37 Chełmońskiego Street, 51-630 Wrocław, Poland; piotr.juszczyk@upwr.edu.pl

**Keywords:** *Dipsacus fullonum* L., irydoids, polyphenols, UPLC-PDA-QTof-MS/MS, anti-bacterial, anti-yeast, anti-oxidant, anti-acetylcholinesterase activities

## Abstract

The aim of the study was to identify and evaluate the content of iridoids and phenolic compounds in the leaves and roots of *Dipsacus fullonum* L. They were identified and quantified by UPLC-PDA-MS/MS. Five iridoid compounds (loganic acid, loganin, sweroside, cantleyoside, and sylvestroside III) were identified in *Dipsacus fullonum* L. leaves and roots. Seven phenolic acids and three flavones were identified in the leaves, and seven phenolic acids were detected in the roots. The leaves contained more iridoids and phenolic compounds than the roots. We also evaluated the antimicrobial (anti-bacterial and anti-yeast), antioxidant (ORAC methods), and antiacetylcholinesterase (AChE) activities of *Dipsacus fullonum* L. leaves and roots. Leaf extract demonstrated the strongest antioxidant activity, but roots showed stronger antiacetylcholinesterase activity than leaves. The study also confirmed antibacterial activity of root-derived compounds against *Staphylococcus aureus* DSM 799 and *Escherichia coli* ATCC 10536.

## 1. Introduction

With a long history of cultivation, consumption, and trade, the everlasting quest for health-promoting and disease-preventing agents in the developed world has changed people’s view of plant sources. Plants have been globally utilized since antiquity for flavoring, coloring, and preserving foods and also for food production, cosmetic, and medicinal purposes [1]. Traditional medicine uses roots and leaves as they are rich in bioactive compounds and excellent sources of compounds such as triterpenes, iridoids, xanthones, carotenoids, and benzophenones [2,3,4]. Polyphenolic compounds such as phenolic acids and flavonoids are still among the crucial compounds exhibiting high antioxidant activity. They are common plant secondary metabolites necessary not only in plant physiological processes but also exerting positive effects on human health as antioxidants. Biological properties of these compounds were demonstrated in vitro and in vivo and they include antioxidant [5], antifungal, antimicrobial [6], antidiabetic [7], anti-inflammatory [5,8], antipyretic, analgesic, and immunomodulatory activity [2].

Apart from phenolics, other compounds such as iridoids have attracted increasing attention. Iridoids belong to bicyclic monoterpenes with a cyclopentane-pyran ring in their structure, and they mostly exist in plants as iridoid glycosides, linked through β-hemiacetal bond to C1. Basic iridoids isolated from plants are loganic acid, loganin, secologanin, sweroside, oleuropein, and pentoside of loganic acid. Iridoids are biologically active compounds with different activities. Their anti-inflammatory, neuroprotective, hypotensive, antibiotic, and sedative properties depend on their structure [9,10,11]. A hydrolyzed product of the iridoids, harpagoside andharpagide, more effectively inhibits cyclooxygenase-2 than the other natural metabolites. Plants rich in this type of monoterpene include Apocynaceae, Lamiaceae, Loganiaceae, Rubiaceae, Scrophulariaceae and Verbenaceae [12]. The compounds are found mainly in the leaves and young stems and hardly ever in fruits. Some iridoids were identified in the leaves of *Sambucus ebulus* [13] and in *Penstemon barbatus, Castilleja tenuiflora, Cresentia alata*, and *Vitex mollis* [11].

The referenced studies reflect the increasing interest in finding plant resources with high biological activity for scientific research and industrial applications.

*Dipsacus fullonum* L. is a flowering plant commonly known as teasel or wild teasel. It is a 1–2.5 m tall, herbaceous, biennial plant with prickly stems, a distinctive cone-shaped flower head, and wrinkled, downward-pointing prickly leaves. *D*. *fullonum* has been used to treat several diseases, like fibromyalgia, bone fracture, lime disease, and especially cancer and Alzheimer’s disease [14,15]. Additionally, it has cytoprotective properties, is capable of inhibiting HIV-1 reverse transcriptase, and may act as an antibacterial, anti-inflammatory, and anti-complement agent, as well as a growth stimulant in osseous cells [14,15]. Root extracts of *D. sylvestris,* a plant belonging to the Dipsacus genus, were active against *Borrelia burgdorferi* s.s., which causes Lyme disease [14,16]. The plant roots of *D. ferox* and *D. asperoides* were reported to contain iridoid glycosides and phenolic compounds [17,18,19]. However, there are no data on the presence of bioactive compounds in *D. fullonum* leaves, and no information is available also about antioxidants and antidiabetic potential or antibacterial activity.

Therefore, the aim of this study was to identify and determine the content of iridoids and polyphenolics in the leaves and roots of *Dipsacus fullonum* L. by means of UPLC-PDA-QTof-MS/MS and to evaluate their biological activity, especially antioxidant, anticholinesterase, antidiabetic. The potential toxicity of the leaves and roots extracts towards microorganisms was evaluated using the antimicrobial test carried out against bacterial strains (as *Bacillus subtilis B5*, *Escherichia coli* ATCC 10536, *Pseudomonas aeruginosa* DSM 939, *Pseudomonas fluorescens* W1, *Staphylococcus aureus* DSM 799) and against selected yeast (*Candida famata* AII4b, *Candida tropicalis* ATCC 60557, *Candida sphaerica* FII7A, *Saccharomyces cerevisiae* SV30, *Yarrowia lipolytica* PII6a). To our knowledge, this is the first report comparing polyphenolic and iridoid compounds and bioactivity of the leaves and roots.

## 2. Results

### 2.1. Identification of Iridoids and Phenolics in D. fullonum L. Leaves and Roots

Table 1 shows qualitative identification of the compounds found in *D. fullonum* L. leaves and roots. In analyzed plant materials 15 compounds belonging to two groups were identified—monoterpenes (iridoids) and polyphenols (flavons and phenolic acids).

UPLC-PDA-QTof-MS/MS for monoterpenes singled out five compounds as iridoids, two of which were positively identified by comparison with standards if they available and the others were tentatively described by their spectra of individual spectral data of peaks (UV/Vis, MS), elution order, UPLC retention times, and by comparison with literature data.

Peak **1** corresponded to characteristic molecular formula of C_16_H_24_O_10_. After deprotonated molecule the ion at *m*/*z* 213 was found after loss of glucose (162 Da). Additionally, comparison with a standard peak 1 was identified as loganic acid.

Peak **2**, with a [M − H]^−^ ion at *m*/*z* 389, and in the MS/MS spectrum with the ions at *m*/*z* 227 that were yielded via a neutral loss of glucose (162 Da) from the deprotonated molecule [M − H]^−^ of peak 2, was identified as loganin based on comparison with a reference compound.

Peak **3** had a major fragment ion at *m*/*z* 195 indicating the presence of a glucose unit (162 Da) in its structure. By comparison with the available standard and reference [20], this compound was tentatively identified as sweroside.

Peak **4** after a loss of glucose unit (162 Da) presented main mass as [M − H]^−^ ion at *m*/*z* 745, and *m/z* 583. This compound was tentatively identified as cantleyoside, which is in agreement with literature data [19].

Peak 5 presented the characteristic of a [M − H]^−^ ion at *m*/*z* 583, and *m*/*z* 421 after loss of glucose (162 Da). After comparison to the literature [15,21,22] this peak was identified as sylvestroside III.

All analyzed leaves and roots contained the same types of compounds. Some were previously identified in other plants, such as berry of blue honeysuckle [23] and fructus Corni [24]. The roots of *D. asper* were previously reported to contain such iridoid glucosides as loganin, lisianthioside, cantleyoside, triplostoside A, or 6-*O*-β-d-apiofuranosylsweroside [23], as well as loganic acid ethyl ester, loganin, cantleyoside and syringaresinol-4′,4′′-*O*-bis-β-d-glucoside [25,26]. The extent and content of iridoid compounds may vary relying on the growth period, year of harvest, geographic location, or cultivation method [27].

*D. fullonum* L. leaves and roots contained seven derivatives of phenolic acids, i.e., caffeoylquinic acid derivatives (Table 1). Four of them revealed the same [M − H]^−^ at *m*/*z* 353 and λ_max_ = 325 but it was derived from different compounds, i.e., neochlorogenic (**1**), chlorogenic acid (**2**), cryptochlorogenic (**3**), and one caffeoylquinic (**4**) acid. In addition, three di-caffeoylquinic acids (**8**–**10**) were identified with [M − H]^−^ at *m*/*z* 515 with λ_max_ = 326. Cochromatographic standards for neochlorogenic, chlorogenic, cryptochlorogenic, and 3,5-dicaffeoylquinic acid were used to confirm the identity of these compounds. All of them were previously reported by other authors [15,20,28]. Caffeoylquinic acid is a phenolic acid very common in different parts of plants, especially 5-caffeoylquinic acid (chlorogenic acid). Analyzed *D. fullonum* leaves contained three flavons identified as apigenin and luteolin derivatives, according to their UV spectrum and MS fragmentation (Table 1). Roots contained only one of these flavons. Two peaks were identified as apigenin derivatives, -6-*C*-hexoside-7-*O*-hexoside (saponarin -*m*/*z* 593 and with λ_max_ = 334 nm) and -6-*C*-hexoside (saponaretin -*m*/*z* 431 and with λ_max_ = 339 nm). All these compounds exhibited MS/MS as [M–fragments of *C*-hexoside]^−^ *m*/*z* 311 typical of *C*-hexoside derivatives. The second flavon was a single luteolin derivative with MS/MS as characteristic [M−H]^−^ *m*/*z* 447 and MS-. MS fragmentation *m*/*z* 327 with λ_max_ = 347 nm of luteolin 8-*C*-β-d-glucopiranoside (orientin). The presence of some of these compounds was also suggested by Kowalczyk et al. [29] and Yang et al. [30].

### 2.2. Quantification of Iridoid and Phenolic Compounds in D. fullonum L. Leaves and Roots

Iridoid content is one of the most important indicators of biological activity in *D. fullonum* L. leaves and roots. Table 1 shows the content of iridoids in the investigated organs—leaves (two study years) and roots (one study year). They were the most abundant in the leaves collected in the first year (41.09 mg/g dry weight—dw), and in the second year their content dropped by 22% and 19% in the leaves and roots, respectively. The content of individual iridoids was highly variable. The major iridoid in *D. fullonum* L. roots was cantleyoside that accounted for 64% of total iridoids, and the second was loganic acid that amounted to 16% of all compounds. The remaining iridoids in the root were sylvestroside III (10%), loganin (9%) >> sweroside (1%). A dominant iridoid in *D. fullonum* L. leaves was sylvestroside III that accounted for 85% of total iridoids in the leaves collected in 2017 and 60% in the leaves from 2018. Abundance of the other iridoids in the leaves was 6% and 37% in 2017 and 2018 for cantleyoside > 6% and 1% for loganic acid >> 2% and 1% for sweroside and for loganic. These results indicate that *D. fullonum* L. leaves collected in 2017 and 2018 may be, apart from commonly used roots, a useful source of biologically active compounds, such as iridoids.

High content of iridoids is beneficial, as they exhibit considerable biological activity. For example, loganic acid has strong anti-inflammatory properties [11]. In rabbits [31], loganic acid exhibited anti-inflammatory activity, diminished diet-induced dyslipidemia and atherosclerosis, and increased PPAR-a and PPAR-expression.

The observed differences between years are probably due to weather conditions in the harvest season and a degree of plant maturity, as suggested by Dinda et al. [9], Pieri et al. [13], and Yang et al. [32]. Kucharska et al. [27] showed that average content of iridoids in cornelian cherry, ranging from 86.91 to 493.69 mg/100 g fresh weight, strictly depended on cultivar and genotype, and the main compound was loganic acid.

Moreover, as presented in Table 1, *D. fullonum* leaves seem to be a much richer source of biologically active polyphenolic compounds than roots. The derivatives of apigenin and luteolin were only found in the leaves, and they were more abundant in those collected in 2017 than in 2018 (16.18 vs. 7.33 mg/g, respectively). Derivatives of caffeic acid were detected in both leaves and roots (35.4 mg/g vs. 16.09 mg/g, respectively) and were the most abundant in the leaves collected in 2018. Chlorogenic acid, known for its considerable biological activity, was a dominant phenolic acid in all samples. Shin et al. [33] reported that phenolic acids, especially chlorogenic acid, had potent antioxidant capacity and may inhibit DNA damage or increase the resistance of LDL to lipid peroxidation. These properties help to protect the human body against chronic non-communicable diseases, including obesity and diabetes. High concentration of chlorogenic acid is typical for fruits and their products [34]. Tandon et al. [35] showed that leaf and root extracts of *A. parviflora*, *A. bracteosa*, and *T. quadrifarium* contained such phenolic acids as protocatechuic, *p*-hydroxybenzoic, chlorogenic, and vanilic. Oszmiański and Wojdyło [36] detected only traces of phenolic acid derivatives (ellagic and gallic) and flavan-3-ols in the roots of *Aruncus silvester* and found that the roots of *Potentilla alba*, *Geumrivale*, and *Waldsteiniageoides* were rich in tannins but not in phenolic acids.

Previous studies demonstrated that the leaves of different berries (such as blackcurrant, bilberry, cranberry, and chokeberry) contained significantly more polyphenols than the fruits [34,37]. *D. fullonum* leaves collected in 2017 and 2018 contained about two and four times more total phenolics than roots. Moreover, Paliyath et al. [38] suggested that phenolic content in the leaves might vary depending on such factors as drought, temperature changes, pollution, UV light, and pathogen attack. Tattini et al. [39] confirmed a significant effect of these factors on the pool of antioxidants in *Ligustrum vulgare* leaves. Additionally, Wang and Lin [40] also suggested that polyphenol content in tissue might determine the degree of plant maturity.

### 2.3. Antimicrobial Activity

Various fungal, bacterial, and viral species could cause plant, animal, and human diseases, hence the diseases of crops, food spoilage, or even food poisoning that could damage human health [38]. Thereby, it is important to extend natural effective antimicrobial agents. Our study analyzed antimicrobial activity of leaves and roots of *D. fullonum.* No information has been available on the antibacterial activity of this species. The extracts of *D. fullonum* leaves and roots were tested against selected bacteria *B. subtilis* B5, *E. coli* ATCC 10536, *P. aeruginosa* DSM 939, *P. fluorescens* W1, *S. aureus* DSM 799, and yeast species: *C. famata* AII4b, *C. tropicalis* ATCC 60557, *C. sphaerica* FII7a, *S. cerevisiae* SV30, and *Y. lipolytica* PII6a. The well diffusion test showed that *E. coli* ATCC 10536 and *S. aureus* DSM 799 were the most sensitive to root extract, and they were the only strains for which a zone of growth inhibition around the well was observed following treatment with the root extract.

**Figure 1:**
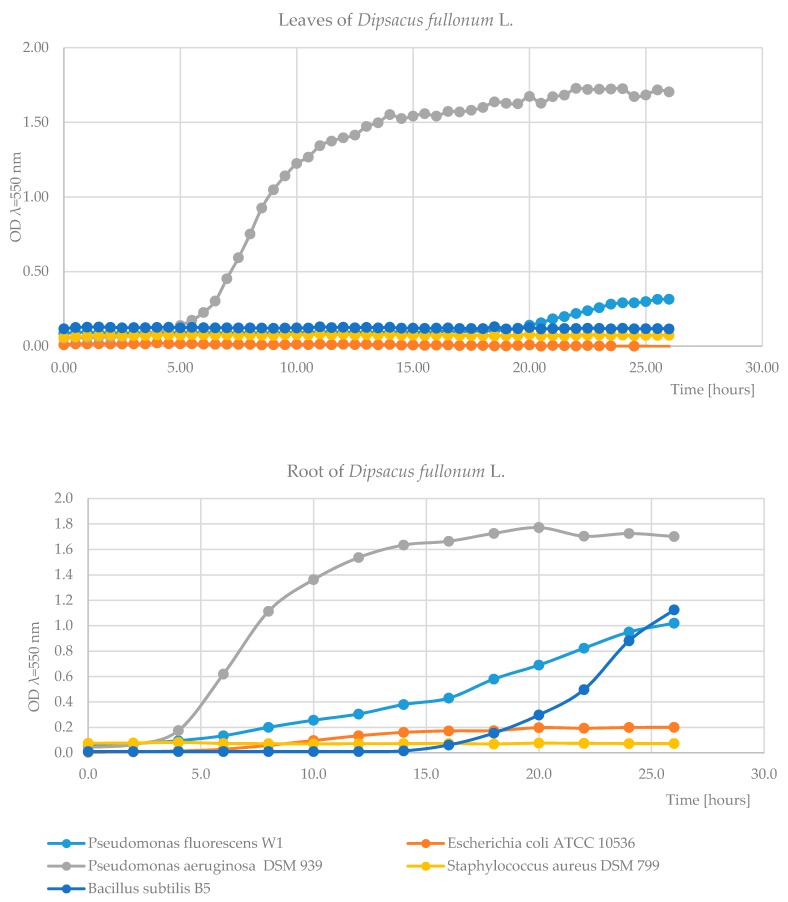
Effect of extract of leaves (**top**) and root (**below**) on bacterial growth in a micro-culture in a Bioscreen C apparatus (*t* = 26 h, T = 30 °C).

Our results were cross-checked by the spot test—zones of inhibited growth of *E. coli* ATCC 10536 and *S. aureus* DSM 799 were found around dried root powder. We found no other positive or negative effects of plant materials in other tested bacteria and yeast. Taking these results into account, the effects of root and leaf extract on bacterial growth were assessed during cultivation in the Bioscreen C analyzer (Figure 1). The test showed significant differences in the bacterial growth depending on the strain and plant material. Optical density (OD) ranged from 0.1 to 1.75. After 26 h of the experiment, the highest OD of 1.8 was observed in the culture of *P. aeruginosa* DSM 939 in medium containing aqueous leaves and root extracts. Based on the growth curves in control NB-medium of the tested bacteria, no effects (positive and negative) of extracts of *D. fullonum* L. on microbial growth was found. In more sensitive cells of *B. subtilis* B5 and *P. fluorescens* W1, OD values ranged from 1.0 to 1.2 but only in medium with root extract. The most conspicuous effect was observed for *E. coli* ATCC10536 and *S. aureus* DSM 799. The tested leaves and root extracts showed a strong antibacterial activity, with OD 0.1 and 0.2, respectively. Based on the growth curves of the tested bacteria, no effects (positive or negative) of extract of *D. fullonum* L. leaves on microbial growth were found. Stanković et al. [41] evaluated different plant materials for their antimicrobial activity. They tested methanolic extracts against pathogenic bacteria isolated from human material. The extract from *Laseritiumlati folium* had the lowest inhibitory concentration against all the tested bacterial strains, i.e., *E. coli, S. aureus*, *P. aeruginosa,* and others. The extract from *Angelica pancicii* showed the strongest antimicrobial activity against *S. pyogenes* and *P. aeruginosa* and the extract from *Angelica sylvestris* against *Streptococcus pyogenes* and *P. aeruginosa*. The main factors molding the antimicrobial activity of plants involve the structure of main bioactive compounds and type of pathogen. In general, bacteria like gram-positive (i.e., *Bacillus* sp. and *Staphylococcus* sp.) are more sensitive than gram-negative ones (i.e., *Escherichia* and *Pseudomonas*) because their membranes are rich in lipopolysaccharides and form an impassable barrier to the bioactive compounds [42]. Additionally, antimicrobial activity is due to the presence of such bioactive compounds as polyphenolic [43,44,45,46] and essential oils as suggested by Aghraz et al. [47]. The antibacterial activity of flavonoids is well documented. Osawa et al. [43] estimated the activity of several different flavonoid compounds. They postulated that chalcones are more effective than flavanones or flavones, and the presence of hydroxyl groups at 2′ position are important for anti-staphylococcal activity of these compounds [43]. When methoxy groups were present, the antibacterial activity of flavonoids significantly reduced. Ohemeng et al. [44] tested 14 compounds belong to flavonoids, among which apigenin, quercetin, and 3,6,7,3′,4′-pentahydroxyflavone presented some inhibitory activity against bacteria such as *Escherichia coli* DNA gyrase and *S. epidermidis*, *S. aureus*, *S. typhimurium*, and *Stenotrophomonas maltophilia*. Bernard et al. [45] found that a glycosylated flavonol, quercetin-3-*O*-rutinoside, exhibited strong antibacterial activity against a permeable *E. coli* strain. Seow et al. [46] postulated that not only the main compounds but also trail compounds are responsible for antimicrobial activity. Nevertheless, the lack of antimicrobial function of some iridoid and polyphenolic compounds in *D. fullonum* leaves and roots does not exclude their pharmaceutical value.

### 2.4. Antioxidant and Antiacetylcholinesterase Activity

Despite their traditional use, leaves are rarely used nowadays, in contrast to berry and fruits [2] or other plant organs such as roots or stems, which are considered food with significant health benefits conveyed by antioxidant activity.

We investigated the antioxidant activity (ORAC) and antiacetylcholinesterase (AChE) activity of leaves (from 2017) and roots. The effects of antioxidant present in the leaves and roots of *D. fullonum* were 14.78 ± 0.94 and 10.87 ± 1.04 mmol/100 g, respectively. High antioxidant activity of plants depends on many factors including the chemical profile of the analyzed material (qualitative and quantitative profiles of bioactive constituents) or plant organs. Our results show that the amount of iridoids weakly correlated with in vitro antioxidant activity measured by ORAC assay. This is due to the structure of iridoids that have none or only limited number of OH groups active in free radical scavenging [48]. Similar to our work, other authors reported poor hydrogen-donating ability of iridoids. Iridoids exhibit high biological activity, especially anti-inflammatory, and perform other biological functions [11,30]. Their antioxidant activity is modulated by phenolic compounds, especially by high content of caffeoylquinic acids.

Nowadays, the search for new sources of effective acetylocholinoesterase inhibitors for the treatment of neurodegenerative disorders such as Alzheimer’s disease is extremely important, as acetylcholinesterase catalyzes hydrolysis of a neurotransmitter acetylcholine that terminates signaling events across cholinergic synapses, including those of neuromuscular functions. Some studies suggest that dietary supplements with antioxidants and free radical scavengers may display benefits in slowing the mild cognitive impairment of Alzheimer’s disease [49]. Our results for anti-AChE enzymes were similar, and the roots of *D. fullonum* L. exhibited stronger inhibitory effect against AChE than leaves (47.14 ± 0.05 vs. 43.89 ± 0.03%, respectively). This effect may be explained, similar to as antioxidant activity, by the presence of biologically active compounds.

Bivar Roseiro et al. [50] reported that flavonoids with a free OH-group at C3 are more effective inhibitors than their C3−OH glycosylated counterparts and those having no C3−OH group, such as luteolin and apigenin. However, the roots we investigated did not contain luteolin or apigenin. Therefore, we suppose the observed effects were due to phenolic acids or iridoids or other chemical constituents, including triterpene saponins and alkaloids identified in the roots of *D. asper* [15]. Other research [49] also shows that many plants rich in natural antioxidants have been proposed as alternative therapeutic agents for Alzheimer’s disease. Ji et al. [26] showed that some iridoids isolated from the roots of *D. asper* exhibited a moderate neuroprotective activity against Aβ_25-35_-induced cell death in PC12 cells.

## 3. Materials and Methods

### 3.1. Reagents and Standards

Acetonitrile, formic acid, methanol, loganin, and loganic acid were purchased from Sigma-Aldrich (Steinheim, Germany). Chlorogenic, neochlorogenic, cryptochlorogenic, 3,5-dicaffeoylquinic acids, and luteolin- and apigenin-7-*O*-glucoside were purchased from Extrasynthese (Lyon, France).

### 3.2. Plant Material

*Dipsacus fullonum* L. roots and leaves from the first and second year of vegetation were obtained from a private garden in Chmęntowo (52°56′02.0″ N 17°46′15.9″ E), Pomerania, Poland. The raw material was collected in October 2017 (leaves and root) and 2018 (leaves). The leaves and roots were dried in a freeze dryer Alpha 1-4 LSC (Christ, Osterode, Germany). Then, they were pulverized by crushing with a closed laboratory mill (A.11; IKA, Staufen, Germany), and obtained powder was kept in a refrigerator (−80 °C) until the extract preparation.

### 3.3. Extraction Procedure for Polyphenolic, Antioxidant as ORAC Method, and Anti-Acetylcholinesterase Activity Analysis

Dried powdered samples of roots and leaves were extracted with 50% methanol. The extraction was performed twice via an incubation for 20 min under sonication with shaking (Sonic 6D, Polsonic, Warsaw, Poland), then the sample was centrifuged at 19,000× *g* for 10 min, and the supernatant was filtered through a hydrophilic PTFE 0.20 μm membrane (Millex Samplicity Filter, Merck, Darmstadt Germany) before analysis. The content of iridoids and polyphenolics was determined by means of ultra-performance liquid chromatography-photodiode array detector-mass spectrometry (UPLC-PDA-QTof-MS/MS) method. All extractions were carried out in triplicate.

### 3.4. Identification and Quantification of Iridoids and Phenolic Compounds by the UPLC-PDA-QTof-MS/MS Method

An ACQUITY Ultra Performance LC system (Waters Corp., Milford, CT, USA) equipped with a photodiode array detector (PDA), a binary solvent manager, and a mass detector G2 Q-Tof micro mass spectrometer (Waters Corp., Manchester, UK) equipped with an electrospray ionization (ESI) source operating in negative and positive mode were used for identification and quantification of polyphenols and iridoids compounds [33,35,51,52]. Column 1.7 µm, 2.1 × 100 mm type UPLC BEH C18 column (Waters Corp., Milford, CT, USA) was used for separating compounds. For analysis, 10 µL of sample was injected, and the elution was completed in 15 min at 30 °C with a sequence of linear gradients and isocratic flow rates of 0.42 mL min^−1^ of A (0.1% formic acid, *v*/*v*) and solvent B (100% of acetonitrile) solvents: 0–12 min from 99% to 65% of A, 12.1–13.5 min from 65% to 0% of A, then gradient was returned to 99% of A solvent to re-equilibrate the column for 15 min. The mass spectrometer was performed within ESI−MS accurate mass experiments operated in negative-ion mode, set to the base peak intensity (BPI) chromatograms with scanning from *m*/*z* 100–1500 and the lock mass correction ±1.000 for the mass window. The optimized MS conditions were as follows: Cone voltage of 30 V, capillary voltage of 2500 V, argon as collision gas, with voltage ramping cycles from 0.3 to 2 V, desolvation and source temperature were 300 and 100 °C, respectively. Leucine enkephalin at a concentration of 500 pg/μL with [M − H]^-^ ion at 554,2615 Da was used as the reference compound. The data obtained from LC−MS were subsequently entered into the MassLynx 4.0 ChromaLynx Application Manager software (Waters Corp., Milford, CT, USA). The PDA spectra were measured over the wavelength range of 200−800 nm in steps of 2 nm. The calibration curves were made for the standard loganin (*y* = 35595*x* − 64514; *r*^2^ = 0.9995) and loganic acid (*y* = 27901*x* + 108708; *r*^2^ = 0.9994), (at 240 nm), for chlorogenic (*y* = 26754*x* + 172359; *r*^2^ = 0.9995), neochlorogenic (*y* = 30401*x* + 91716; *r*^2^ = 0.9994), cryptochlorogenic (*y* = 30726*x* + 190297; *r*^2^= 0.9976), 3,5-dicaffeoylquinic acids (*y* = 39233*x* − 360853; *r*^2^ = 0.9994) (at 320 nm), and for luteolin-7-*O*-glucoside (*y* = 11211*x* + 498134; *r*^2^ = 0.9835), apigenin-7-*O*-glucoside (*y* = 20768 + 121323*x*; *r*^2^ = 0.9912) (at 340 nm) at concentrations ranging from 0.05 to 0.5 mg/mL. Loganin and loganic acid were expressed as loganin and loganic acid, respectively, but the rest of the iridoids (sweroside, cantleyoside, and sylvestroside III) were calculated as loganin compounds. Neochlorogenic acid was expressed as neochlorogenic acid, chlorogenic acid was expressed as chlorogenic acid, cryptochlorogenic acid was expressed as cryptochlorogenic acid, and 3,5-dicaffeoylquinic and caffeoylquinic acids and their isomers were expressed as 3,5-dicaffeoylquinic acid. Apigenin compounds were expressed as apigenin-7-*O*-glucoside, and luteolin -*C*-D-glucopiranoside was expressed as luteolin-7-*O*-glucoside. The results were expressed as milligrams per g dw.

### 3.5. Antimicrobial Activity

In vitro antimicrobial studies were carried out against 5 bacterial strains, namely *Bacillus subtilis* B5, *Escherichia coli* ATCC 10536, *Pseudomonas aeruginosa* DSM 939, *Pseudomonas fluorescens* W1, *Staphylococcus aureus* DSM 799. Additionally, activity of extract was analyzed against selected yeast: *Candida famata* AII4b, *Candida tropicalis* ATCC 60557, *Candida sphaerica* FII7a, *Saccharomyces cerevisiae* SV30, *Yarrowia lipolytica* PII6a. All bacterial strains and yeast were from the collection at the Department of Biotechnology and Food Microbiology, Wrocław University of Environmental and Life Sciences. Dried powdered samples of roots and leaves were extracted with aqueous solution. The extraction was performed twice via an incubation for 20 min under sonication with shaking (Sonic 6D, Polsonic, Warsaw, Poland), then sample was centrifuged at 19,000 × *g* for 15 min, and the supernatant was used for analysis. Extractions were carried out in triplicate.

#### 3.5.1. Agar Diffusion Method: The Effects of Water Extracts from Leaves and Root on Growth of Bacteria and Yeast

Inoculum preparation. Each bacterial strain was sub-cultured in medium NB (Merck, Darmstadt, Germany) at 30 °C for 24 h. After centrifugation (10,000× *g* rpm; 20 min; Isolab D2012, Laborgeräte GmbH, Eschau, Germany), bacterial cells were washed twice, using sterile water, and suspended in fresh sterile NB medium. Absorbances were adjusted at 620 nm and diluted to attain viable cell count of 1 × 10^9^ cfu/mL using a spectrophotometer (Smart Spec Plus, Biorad, Hercules, CA, USA) (optical density, OD_620_ = 1.5)

Yeast were cultured in the YM broth, which consisted of 3 g yeast extract, 3 g malt extract, 5 g bacteriological peptone, and 10 g of glucose dissolved in 1 L of distilled water. The cultivations were conducted for 48 h in 0.3 L flasks containing 0.075 L medium on a rotary shaker (G10 Gyrotory Shaker, NewBrunswick-Scientific Co.; Edison, NJ, USA) at 160 rpm and 25 °C. After centrifugation (7000 rpm; 20 min; Isolab D2012, Laborgeräte GmbH, Eschau Germany), yeast cells were washed two times and suspended in fresh sterile YM broth. The suspension was standardized with 2 McFarland turbidity standard (McFarland Standard, bioMerieux, Marcy l’Etoile, France) equal to 1 × 10^6^ cfu/mL.

One mL of bacterial suspension (1 × 10^9^ cells/mL) and 1 mL of yeast suspension (1 × 10^6^ cells/mL) were mixed with 20 mL melted (45 °C) NB-agar and YM-agar, respectively, and poured into Petri dishes.

The plates were kept in the fridge at 5 °C for 2 h. To permit plant extract diffusion, they were then incubated at 30 °C for 2–7 days. The examinations were carried out in triplicate. The presence of inhibition (−) or intensive growth (+) zones were measured by Vernier caliper, recorded, and considered as indications of antibacterial activity. No effect was marked as (0).

#### 3.5.2. Well Diffusion Method

Well diffusion method on NB-agar (Merck) was used for evaluating antibacterial activities of water extract from root and leaves. Under aseptic conditions, NB agar plates were inoculated by tested bacterial or yeast strain. Test samples (50 μL) were added to each well with 6 mm diameter holes and incubated for 24–72 h at 30 °C. After this period, confluent bacterial or yeast growth was observed. The following control agents were positive control agents—oxytetracycline (5 µg/mL) (for bacteria) and cycloheximide 20 mg/mL (for yeasts).

#### 3.5.3. Spot Test

The powder from leaves and roots of D. *fullonum* L. were spread onto the surface of the above media (0.05 mg) using a sterile spatula. As a positive control, oxytetracycline crystals (bacteria) and cycloheximide (yeast) crystals were used.

#### 3.5.4. Growth in Bioscreen C Microbial Growth Analyzer

The tests were performed in the automated Bioscreen C system (Automated Growth Curve Analysis System, Lab systems, Helsinki, Finland) according to the procedure described by Wróblewska et al. [52]. NB medium and water extracts from plants were filter sterilized using sterile, disposable syringe filters of 0.2 mm pore size 13 mm diameter. The bacterial inoculum was diluted in sterile deionized water to give a concentration of approximately 2° McFarland. The working volume in the wells of the Bioscreen plate was 0.35 mL, comprising 0.30 mL of culture medium with water plant extract and 0.05 mL of inoculum (final concentration 1 × 10^6^ cells/mL). The temperature was controlled at 30 °C, and the optical density of the cell suspensions was measured automatically at 560 nm in regular intervals of 30 min for 50 h. The cell cultures were automatically continuously shaken. Each culture was performed in 5 replications.

### 3.6. Biological Activity

The ORAC assay was determined following the method previously described by Ou et al. [53]. All samples were assayed in triplicate, and the results were expressed as mmol of Trolox per 100 g of dw. The anti-acetylcholinesterase activity was measured as inhibition of acetylcholinesterase as reported previously by Wojdyło, Nowicka, and Bąbelewski [51]. All samples were performed in triplicate and the result was presented as % of inhibition.

### 3.7. Statistical Analysis

Statistical analyses were performed with Statistica version 12.0 software package (StatSoft Inc., Tulsa, OK, USA). All data were presented as mean ± standard deviation of three independent determinations and were subjected to a one-way analysis of variance (ANOVA) by Duncan’s test, while post hoc test was used to compare the mean values.

## 4. Conclusions

The study provides a comprehensive summary of a specific bioactive profile of *Dipsacus fullonum* L. leaves and roots. In the leaves, the most abundant iridoids were cantleyoside and sylvestroside III, and in the roots cantleyoside and loganic acid were prevailing. Phenolic acid, chlorogenic acid, 3,5-dicaffeoylquinic acid, and apigenin-6-*C*-glucoside-7-*O*-glucoside were evenly distributed within the plants. Additionally, our analysis revealed that the leaves collected in the second year of vegetation contained two times more polyphenols than iridoids as compared with the first year. Finally, the roots contained more iridoids than leaves. Leaf extract showed stronger antioxidant activity, but roots exhibited higher antiacetylcholinesterase activity than leaves. Antibacterial activity against *Staphylococcus aureus* DSM 799 and *Escherichia coli* ATCC 10536 was only noted in root extract.

The study demonstrated that *Dipsacus fullonum* L. could be considered a natural source of bioactive compounds, particularly phenolics and iridoids, and confirmed its high potential for the pharmaceutical industry. Future studies focused on nutritional value and technological possibilities of using *Dipsacus fullonum* L. seem well deserved.

## Figures and Tables

**Table 1:** LC-QTOF/MS analysis and quantification of iridoid and polyphenolic compounds (mg/g dry weight—dw) in D. *fullonum* L. leaves and root.

Peak No.	Compound	T_R_ (min)	λ_max_ (nm)	MS *(m*/*z)*	MS/MS (*m*/*z*)	Leaves		Roots
						2017	2018	
**Iridoids**								
1	Loganic acid	3.86	240	375	213/191/169/151/119	2.60 ± 0.11b	0.18 ± 0.06c	5.27 ± 0.67a
2	Loganin	5.18	238	389	227/209	0.64 ± 0.02b	0.56 ± 0.02b	3.02 ± 0.23a
3	Sweroside	5.30	238	357	195/125	0.67 ± 0.11a	0.35 ± 0.01b	0.45 ± 0.06b
4	Cantleyoside	8.24	236	745	583	2.38 ± 0.21c	11.83 ± 1.11b	21.41 ± 2.13a
5	Sylvestroside III	9.14	240	583	421	34.80 ± 3.21a	19.14 ± 3.21b	3.17 ± 0.66c
Total iridoids						41.09	32.06	33.32
**Polyphenol**								
1	Neochlorogenic acid	3.54	325	353	191	0.52 ± 0.1a	0.48 ± 0.05a	0.24 ± 0.02b
2	Chlorogenic acid	5.02	325	353	191	10.98 ± 2.01b	28.44 ± 2.54a	6.47 ± 0.45c
3	Cryptochlorogenic acid	5.27	325	353	191	0.86 ± 0.23a	0.63 ± 0.04a	0.30 ± 0.02b
4	Caffeoylquinic acid	5.56	325	353	191	0.40 ± 0.05b	1.15 ± 0.12a	0.07 ± 0.01c
5	Apigenin-6-*C*-glucoside-7-*O*-glucoside (Saponarin)	5.74	334	593	431/311	5.74 ± 0.12b	8.54 ± 1.43a	0.02 ± 0.01c
6	Luteolin 8-*C*-d-glucopiranoside (Orientin)	5.87	347	447	327	0.42 ± 0.03b	4.65 ± 1.09a	nd *
7	Apigenin-6-*C*-glucoside (Saponaretin)	6.78	339	431	311	1.17 ± 0.12b	2.99 ± 0.38a	nd
8	3,5-Dicaffeoylquinic acid	10.79	326	515	353/191	2.78 ± 0.51b	3.96 ± 0.32a	3.77 ± 0.34a
9	Di-caffeoylquinic acid isomer	11.10	326	515	353/191	0.17 ± 0.11a	0.21 ± 0.03a	0.17 ± 0.02a
10	Di-caffeoylquinic acid isomer	11.45	326	515	353/191	0.38 ± 0.04b	0.53 ± 0.10b	1.39 ± 0.09a
Total polyphenols						23.42	51.58	12.43

* nd—not detected; a, b, c—different letters in the same column denote a significant difference among varieties according to Tukey’s test, *p* < 0.05.
